# Analyzing 6211 unique variants in the upgraded interactive FVIII web database reveals novel insights into hemophilia A

**DOI:** 10.1016/j.bvth.2025.100053

**Published:** 2025-01-21

**Authors:** Emily H. T. Print, Anna M. Simmons, Holly J. Spencer, Christos Efthymiou, Victoria A. Harris, Stephen J. Perkins

**Affiliations:** Research Department of Structural and Molecular Biology, University College London, London, United Kingdom

## Abstract

Hemophilia A is a rare genetic disease that occurs with mild, moderate, or severe phenotypes and involves dysfunctional or reduced amounts of plasma factor VIII (FVIII). Identifying causal genetic variants in the *F8* gene is vital for patient care. Our original interactive MySQL database for FVIII in 2013 presented clinical data on 2014 unique FVIII variants in 5072 patients. Here, we expand our database almost threefold with a new total of 6211 unique FVIII variants in 10 064 patients, spanning 1529 of the 2351 FVIII residues (65%). We have also developed a new full-length FVIII structural model that incorporates both its crystal structure and its disordered B domain, which is not visible in available experimental structures. This enabled the assessment of these variants on FVIII. Of the 6211 unique *F8* variants identified, 730 (12%) were associated with mild phenotypes, 526 (8%) with moderate phenotypes, 2509 (39%) with severe phenotypes, 53 (1%) with multiple severities, and 2393 (40%) with unreported phenotypes. Most variants occurred in the disordered B domain (1281 variants), followed by the A1, A2, and A3 domains (1130, 1071, and 923 variants, respectively) and the C1 and C2 domains (442 and 439 variants, respectively). Inhibitors were associated with 451 variants (7%). Our new structural analyses often revealed changes to the residue solvent surface accessibilities caused by many FVIII variants. The FVIII variant analyses are supported by similar observations in the structurally related FV protein. Our web-accessible FVIII database will enable easier and improved clinical analyses of FVIII genetic variants.

## Key Points

• Our FVIII database now contains 6211 variants from 10 064 patients and includes 1529 (65%) of the 2351 FVIII residues.
• The FVIII upgrade explains the effects of variants on the FVIII protein and enables improved clinical analyses of FVIII genetic variants.

## Visual Abstract

**Figure:**
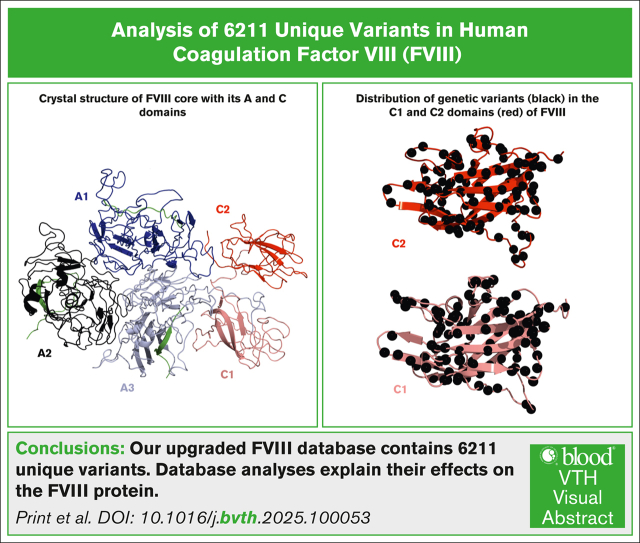

## Introduction

**Figure 1.**
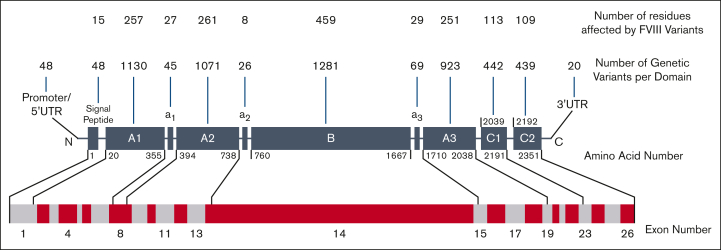
**Domain cartoon of the 6211 unique variants of FVIII.** The FVIII protein comprises the A1-a1-A2-a2-B-a3-A3-C1-C2 domains and linkers and is shown as dark gray boxes that are not drawn to scale. N and C represent the N terminus and C terminus, respectively. Residue numbering marks the first and last amino acids that frame each domain, reported in HGVS format. Above each protein domain, the number of variant residues is shown in the upper row, and the number of variants in each domain is shown in the lower row. Intronic variants are included in their respective domains according to their sequence numbering. Variants that occur in splice sites (420), multiple domains (202), or are not reported (47) are not shown. Below the protein domains, the gene arrangement of 26 exons is shown as alternating light gray and maroon boxes drawn to scale. The exons coding for each FVIII protein domain are indicated. UTR, untranslated region.

Hemophilia A is the most commonly reported bleeding disorder, affecting 1 in 5000 live male births.^1^ It is associated with factor VIII (FVIII; Online Mendelian Inheritance in Man number 300841; gene identification [ID] number 2157), spanning 193 kb across the X chromosome (Xq28).^2^^,^^3^ Its messenger RNA contains 9032 bp (Figure 1). The deduced FVIII protein sequence with a molecular mass of 275 kDa corresponds to 2351 amino acids (UniProt number P00451).^3^ Full-length mature FVIII is arranged as 6 A, B, and C domains with 3 linkers *a1*, *a2*, and *a3* (Figure 1).^2^ Murine and human studies reveal that FVIII is synthesized by the endothelium of multiple organs, including the liver.^4^, ^5^, ^6^, ^7^ Proteolytic cleavage of FVIII by thrombin or activated FX (FXa) occurs after its secretion from endothelial cells,^6^^,^^7^ which leads to the partial removal of the B domain and subsequent secretion into blood plasma. This forms a heterodimer with a heavy chain (A1-*a1*-A2-*a2*-B_partial_) and a light chain (*a3*-A3-C1-C2), which circulates in plasma as a complex with von Willebrand factor (VWF). Upon injury, FVIII becomes activated through further proteolytic cleavage. This results in the release of the B domain and the *a3* linker, causing dissociation of FVIII from VWF. FVIIIa then acts as a cofactor for serine protease–FIXa by forming a complex to catalyze the generation of activated FX. This allows thrombin-driven blood clot formation. The heterotrimer then either dissociates into inactive subunits or is cleaved further by activated protein C to become inactivated.^8^ In hemophilia A, plasma levels of FVIII and/or its functional activity are reduced.^9^ It is classified from its residual FVIII activity (FVIII:C) into mild (5%-40%), moderate (1%-5%), and severe (<1%) phenotypes.^10^ Patients with severe hemophilia A may have 20 to 30 bleeding episodes annually,^9^ although this is not typical of those receiving appropriate prophylactic FVIII replacement therapy or a FVIII mimetic.

Thousands of individual *F8* patient variants have now been reported. Originally, variants were presented as simple listings in the HAMSTeRS (Haemophilia A Mutation, Search, Test and Resource Site).^11^, ^12^, ^13^ Apart from the dedicated CHAMP database from July 2012 with 2537 unique *F8* variants^14^ and the EAHAD Factor VIII Variant Database (which is a copy of our database^15^), we are not aware of other FVIII databases. Other genetic repositories containing *F8* variants include the Exome Aggregation Consortium resource,^16^ the Leiden Open-source Variation database,^17^ the Expert Protein Analysis system, the ClinVar resource, the Genome Aggregation Database (gnomAD) resource, and the Human Gene Mutation Database. Since 2003, the development of searchable web databases for genetic variants in coagulation FXI and FIX provided a more powerful clinical tool to analyze variants in terms of gene and protein sequences, clinical phenotypes, and protein structures.^18^, ^19^, ^20^ The FXI and FIX websites were recently upgraded and supplemented by others for FX and FV.^21^, ^22^, ^23^, ^24^ Our previous FVIII database with 2014 unique variants^15^ and protein structures for 2 FVIII domains^25^ has received >50 500 visits since 2013 when it was replaced with the updated database.

**Figure 2.**
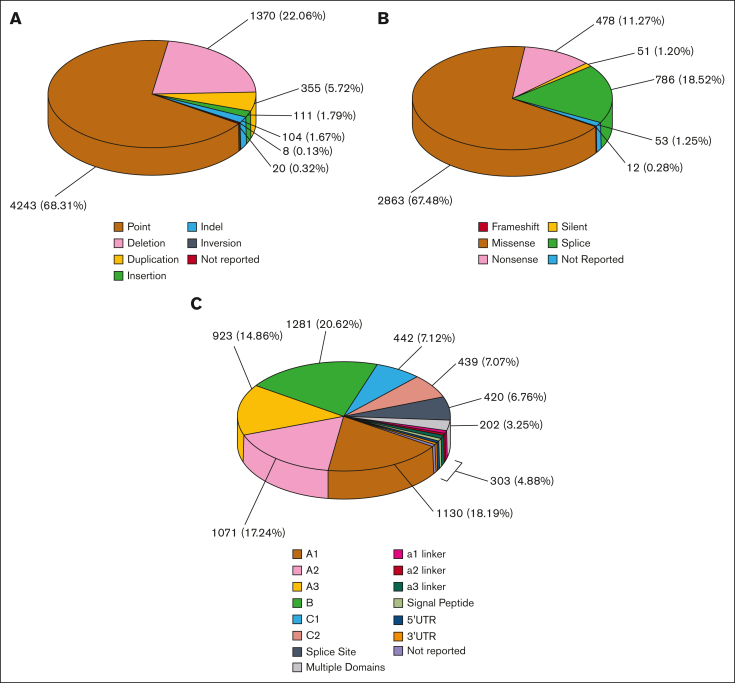
**Distribution of the 6211 unique variants found in the *F8* gene.** Breakdowns of the 6211 FVIII variants into variant type, effect, and location within the *F8* gene sequence are shown. “Not reported” refers to variants found within literature or databases that did not contain any information about the severity of the inherited mutation. (A) The relative frequency of different types of unique variants in the *F8* gene. (B) Effects of the 4243-point variants found in the *F8* gene sequence. (C) Distribution of the 6211 FVIII variants across the *F8* gene and FVIII protein domains.

Here, this major upgrade to our FVIII website almost triples the total of unique FVIII variants to 6211 (Figure 2A), presents a new improved crystal structure for the A and C domains in FVIII (Protein Data Bank [PDB] ID 6MF2),^25^ and reveals a full-length structure prediction for the B domain based on AlphaFold.^26^ The substantial 1529-residue (65%) variant coverage of the 2351 residues in FVIII attributes the damaging effects of many variants to perturbations in the protein structure. The upgraded database significantly improves our understanding of the molecular basis of FVIII deficiency for clinicians and researchers.

## Methods

### Source of the FVIII database

A total of 6211 unique *F8* genetic variants from 10 064 patients with hemophilia A were included in the upgraded FVIII database. The FVIII database was originally created in the same format as our FXI and FIX databases.^18^, ^19^, ^20^ This was based on 2014 unique variants from 5480 patients,^15^ including 1519 variants from the HAMSTeRS listing^11^, ^12^, ^13^ from 3089 patients. The current total of 6211 FVIII variants (from a cut-off date of August 2024) was derived from online literature searches of peer-reviewed articles, primarily PubMed, which was searched using keywords such as “Factor VIII,” “F8,” “hemophilia A,” and “mutation.” BOOLEAN search terms (“AND” and “OR”) were used to refine these keyword searches for existing *F8* publications between 1985 and August 2023. The source publications are listed in the “Reference” tab of the FVIII database. Many variants were also collected through existing genetic database searches, including gnomAD and ClinVar. Data for each variant were first compiled into an Excel spreadsheet, which was then imported into the FVIII MySQL database using phpMyAdmin software (https://www.phpmyadmin.net/) as an intermediary platform. The database is maintained on a University College London server and uses HTML, PHP, and JavaScript programming to enable public access via the web. For personal research use only, a list of the FVIII variants and their associated fields can be downloaded from the “Variants” menu on the website (supplemental Materials).

### Protein structure analysis of the FVIII variants

The FVIII database records DNA and protein changes in Human Genome Variation Society (HGVS) format.^27^ For DNA changes, +1 refers to the A of the ATG initiation codon at the start of the 19-residue signal peptide in *F8*. For protein changes, +1 refers to the ATG initiation codon. Previous publications used legacy numbering instead, which was stipulated as such in the variant comments section. Legacy numbering was converted to HGVS format by adding 19 to the original legacy number (+1 in legacy numbering refers to +20 in HGVS numbering).

Although 21 partial human FVIII crystal structures are available in the PDB and were included on the FVIII database, no structures for full-length unbound FVIII are available. Only 3 crystal structures, all of B domain–deleted FVIII, show the 5 A1, A2, A3, C1, and C2 domains (Figure 1), with ID codes 3CDZ^28^ (resolution, 0.39 nm), 2R7E^29^ (resolution, 0.37 nm), and 6MF2^25^ (resolution, 0.361 nm). A cryo-electron microscopy (cryo-EM) structure for B domain–deleted FVIII bound to the VWF D’D3 domains at 0.29-nm resolution is also available (PDB ID 7KWO).^30^ Here, the most recent unbound 6MF2 structure with the highest atomic resolution was used for variant analyses. The disordered B domain structure was predicted as part of a full-length AlphaFold-modeled FVIII structure (entry ID P00451 from 7 September 2021), similar to previous predictions for FV,^23^ and is very similar to a later model in the Research Collaboratory for Structural Bioinformatics database (entry ID AF_AFP00451F1 from 30 September 2022). AlphaFold is an artificial intelligence system based on machine learning neural networks that predicts protein structures from the sequence, with varying degrees of accuracy.^26^

The Definition of Secondary Structure of Proteins (DSSP) tool (https://www3.cmbi.umcn.nl/xssp/) identified the secondary structure of each residue in the FVIII crystal structure (PDB ID 6MF2) or AlphaFold structure.^25^^,^^31^ These were one of H (α-helix), B (β-bridge), E (extended β-strand), G (3_10_ helix), I (π-helix), T (hydrogen-bonded turn), S (bend), or C (undefined coil region). DSSP was also used to determine the exposed surface area of each FVIII residue in Å^2^. The surface areas were converted into percentage accessibility by dividing the DSSP output by the theoretical solvent-accessible surface area of each amino acid.^31^, ^32^, ^33^ Percentage accessibilities of 0% to 9% were assigned as 0, 10% to 19% as 1, 20% to 29% as 2, and so on. Residues with accessibilities of 0 or 1 were classified as buried and those with accessibilities of 2 to 9 were classified as solvent-exposed. PyMOL was used for visual inspections of the variants in the crystal and AlphaFold structures.

Three substitution analyses assessed the effect of the missense variants on the FVIII structure. These were Polymorphism Phenotyping v2 (PolyPhen-2; http://genetics.bwh.harvard.edu/pph2/),^34^, ^35^, ^36^ Sorting Intolerant From Tolerant (SIFT; https://sift.bii.a-star.edu.sg/www/SIFT_seq_submit2.html)^37^ and Functional Analysis through Hidden Markov Models (FATHMM; https://fathmm.biocompute.org.uk/about.html).^38^ All 3 methods gave reproducible analyses compared with older ones, which reported less clear outputs such as Provean (whose website has been taken down)^39^^,^^40^ and Grantham^41^ (whose results were less clear). For PolyPhen-2 and FATHMM, higher prediction scores closer to 1.0 indicate variants that are more likely to be damaging. For SIFT, prediction scores closer to 0.0 indicate variants that are more likely to be damaging.

## Results

### Classification of FVIII variants in the updated interactive web database

The upgraded FVIII web database in this current study presents 6211 unique genetic variants (Figure 2) from 10 064 patient records, which is almost triple the total of 2014 in the 2013 version.^15^ Variants were sourced from 483 research articles that addressed hemophilia A disease in genotyped patients, this being an increase of 44% from the 336 articles in 2013. Variants were also sourced from web databases (see “Methods”). The database was also updated with interactive features in our upgraded FV, FIX, FX, and FXI websites (see “Introduction”), in which a site map facilitates user navigation.^21^, ^22^, ^23^, ^24^ The website home page featured movies of the AlphaFold-predicted structure and the crystal structure. Allelic frequencies (AFs) were also provided for the FVIII variants when possible, using the gnomAD version 2.1.1,^42^ which spanned 125 748 exome sequences and 15 708 whole-genome sequences. Thus, 1477 (24%) of our 6211 FVIII variants were found in gnomAD. The AF indicated the relative frequency of a given variant at a specific genetic locus. The AF cutoff was taken as 0.01, in which an AF >0.01 denoted a commonly occurring variant. Additional features include a multiple sequence alignment of human FVIII with that of other species, which helps users understand the phylogenetic history of the *F8* gene and the extent of residue conservation in related sequences. If Java applets are enabled within the user’s web browser, this can be accessed via the amino acid alignments tab. An interactive FVIII structure allows for the exploration of the structural and functional impacts of each variant. Lastly, all 21 known FVIII structures and their literature references were presented for analyses in the web database.

**Figure 3.**
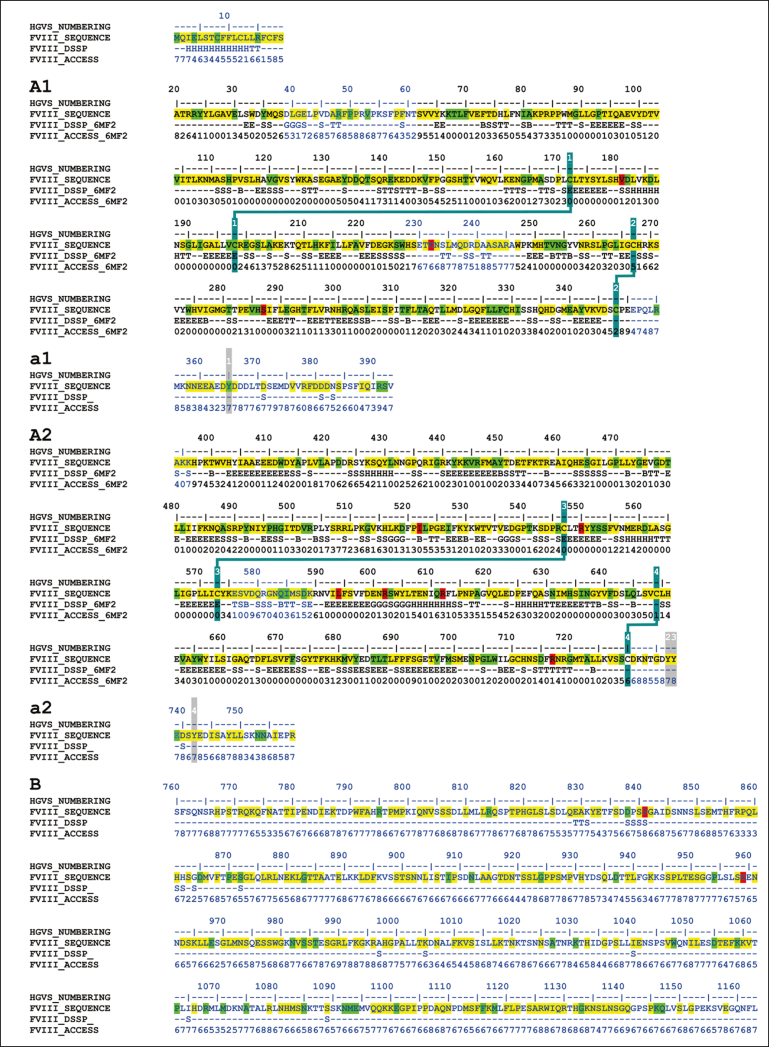

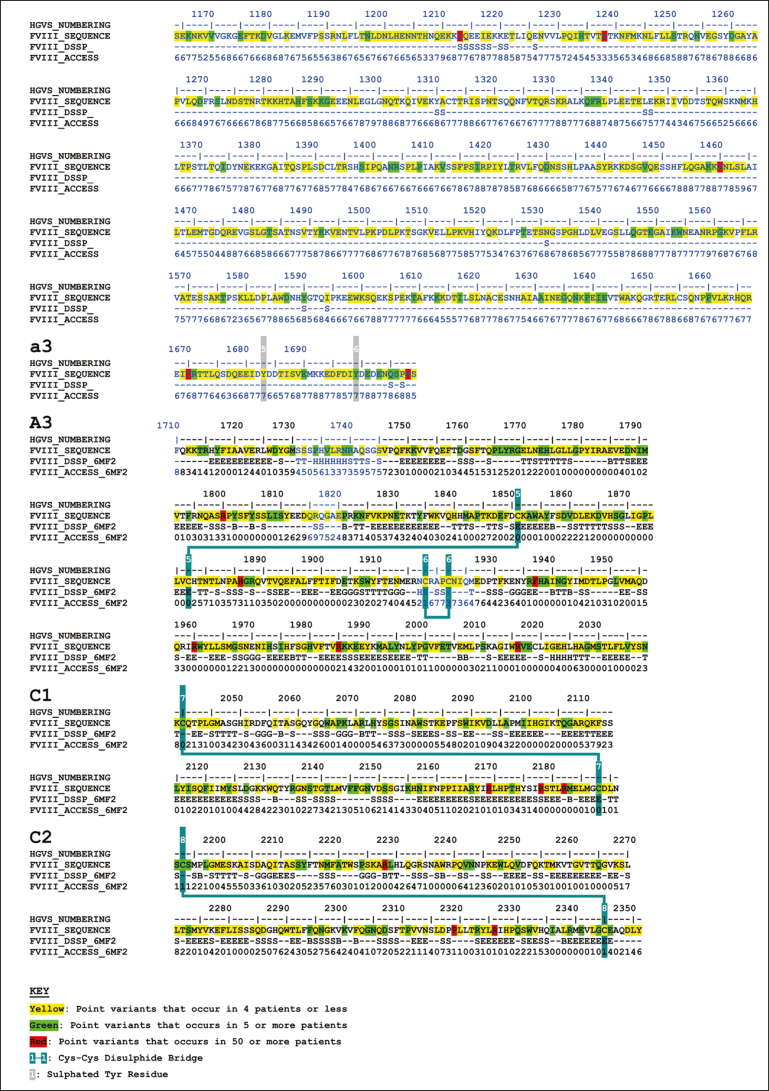
**Secondary structure and accessibility analysis of variants occurring in the FVIII protein.** The FVIII amino acid sequence is shown with secondary structure assignments and solvent accessibilities indicated below each residue. Secondary structures are assigned as H (α-helix), B (β-bridge), E (extended β-strand), G (3_10_ helix), I (π-helix), T (hydrogen-bonded turn), S (bend), or C (undefined coil region). These were determined from the FVIII crystal structure when the residues were visible and present (PDB ID 6MF2). Sequences within this crystal structure are shown in black, and sequences without a crystal structure are shown in blue. For the latter, secondary structure and solvent accessibility predictions were made based on the modeled FVIII AlphaFold structure. The positions of 4243-point variants that occur in the *F8* exons are highlighted in yellow, green, and red. These include point missense, point nonsense, and point silent variants. Yellow denotes point variants that occur in ≤4 patients, green denotes point variants that occur in ≥5 patients, and red denotes point variants that occur in >50 patients. Posttranscriptional modifications are shown. These include 25 putative N-glycan sites (highlighted in cyan), 8 numbered Cys-Cys disulfide bridges (highlighted in blue), and 6 sulfated numbered Tyr residues (highlighted in gray).

Phenotypically, the variants used measurements of the residual FVIII activity (FVIII:C) to determine mild (5%-40%), moderate (1%-5%), and severe (<1%) classifications.^10^ The 6211 variant types included 730 mild (12%), 526 moderate (8%), 2509 severe (39%), 53 (1%) with multiple severities, and 2393 (40%) with an unreported phenotype. Approximately 5% of patients with hemophilia A have normal amounts of dysfunctional FVIII and are termed cross-reacting material positive; this measure was not used in the database because of its infrequent use in the literature. The 6211 variants can also be classified genetically, in which point variants made up 68.3%, deletions 22.1%, duplications 5.7%, insertions 1.8%, indels 1.7%, and inversions 0.1% (Figure 2A). The 4243-point variants corresponded to missense (67.5%), nonsense (11.3%), silent (1.2%), splice site (18.5%), and unreported variants (1.2%; Figure 2B). The variants occurred throughout all 6 FVIII domains and the linker regions (Figure 2C). Several point variants occurred in ≥5 patients in the A and C domains (Figure 3 green highlights). The 8 most common variants (including synonymous variants) occurred in ≥50 patients in all domains (Figure 3, red highlights). These were Leu594Leu (730 patients), Ser287Ser (149 patients), Arg612Cys (138 patients), Arg2169His (103 patients), Gly1238Asp (91 patients), His1886Tyr (83 patients), Arg1985Gln (69 patients), and Arg2178Cys (69 patients; Figure 3).

**Figure 4.**
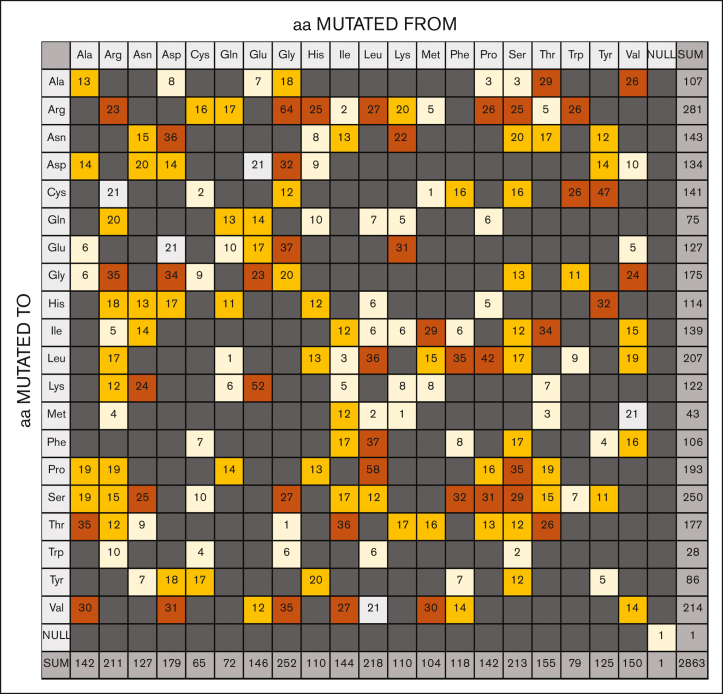
**Substitution grid that summarizes 2863-point missense variants in the *F8* gene.** The grid illustrates the total of missense variants that occurs for each defined amino acid change. All substitutions result from a single nucleotide change. Any grid substitutions that would require more than a single nucleotide change are shown in dark gray, although none were seen. Pale gray represents silent mutations; pale yellow represents substitutions that occur between 1 and 10 times; orange represents substitutions that occur between 11 and 20 times; and brown represents substitutions that occur ≥20 times. aa, amino acid.

Substitution analysis of 2863 FVIII missense variants examined the distribution of the amino acid changes (Figure 4). These substitutions involved only single nucleotide changes, with none in the dark gray boxes, which would indicate 2 nucleotide changes for a substitution. In total, Gly (252 changes), Leu (218 changes), Ser (213 changes), and Arg (211 changes) were the most commonly mutated residues (Figure 4, bottom row). The predominance of positively charged Arg missense variants suggested that ionic interactions were important for FVIII function. The prevalence of hydrophobic Pro, Val, Leu, and Cys variants suggested that the internal FVIII protein structure was disturbed by protein misfolding. The Cys residues were important for forming 2 stabilizing disulfide bridges in the A1, A2, and A3 structures and, similarly, 1 disulfide bridge in each of the C1 and C2 structures (Figure 3). Gly residues with the smallest side chain were the next most frequently mutated, corresponding to the difficulty of accommodating a larger side chain within FVIII. Energetically, glycine can adopt several unique backbone conformations that no other amino acid residue can adopt in an energetically favorable manner. Putative N-linked glycan chains were mostly localized to the B domain (Figure 3), with 2 in the A1 domain, 1 in the A2 domain, 19 in the B domain, and 1 in each of the a3 linker and the A3 and C1 domains.^43^

### Crystal structure analysis of secondary structures

**Figure 5.**
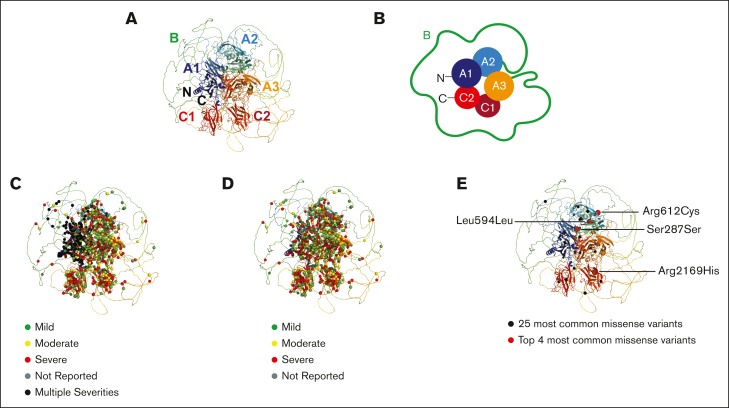
**Structural and schematic views of variants within the FVIII domains.** (A) The AlphaFold model for full-length FVIII is shown in ribbon format. The structure is shown in rainbow colors, starting with blue at the N terminus (N) and ending with red at the C terminus (C). The disordered B domain is depicted as a ribbon encompassing the A and C domains, although its predicted conformation is of very low confidence. (B) The FVIII structure from panel A is shown schematically in cartoon form in the same orientation and colors. The globular A1, A2, A3, C1, and C2 domains are denoted by filled circles. The disordered B domain is schematically represented by a green line. (C) The 2863 missense variants are mapped to the ribbon diagram, in which the occurrence of multiple different variants with mild, moderate, and severe phenotypes are shown as colored spheres. The mild, moderate, and severe variants are overlaid onto the ribbon diagram of panel C. (D) The 2863 missense variants are mapped to the ribbon diagram, in which the phenotype classifications of mild, moderate, and severe effects are denoted as the traffic light colors green, yellow, and red, respectively. (E) The 25 most commonly reported synonymous variants are shown as spheres in the ribbon structure of FVIII shown in panel A. Black spheres denote the 5th to 25th most common variants, and the 4 labeled magenta spheres denote the top 4 most common synonymous variants seen in FVIII.

The 3-dimensional FVIII structure clarified the molecular basis of FVIII variants causing hemophilia A. The 2 earlier FVIII crystal structures used for the 2013 database (PDB IDs 2R7E and 3CDZ)^28^^,^^29^ are superseded by an improved one (PDB ID 6MF2),^25^ in all 3 of which the B domain is not present. The latter structure is a model of human B domain–deleted FVIII, based on a crystal structure of a porcine-human hybrid FVIII with porcine A1 and A3 domains (PDB ID 6MF0). The human 6MF2 structure showed an improved resolution of 0.361 nm compared with 0.398 nm for 3CDZ, which enabled a better analysis of FVIII variants. When subjected to Ramachandran analysis (http://molprobity.biochem.duke.edu), the 6MF2 model gave a better goodness-of-fit R value of 0.252, compared with an R value of 0.256 in the 3CDZ model. The 6MF2 model also had a better free R value of 0.284 compared with 0.327 for the 3CDZ model. In 6MF2, 82% (883 residues) were categorized in the “most favored” conformational regions, 13% (145) were in the “additional allowed” regions, and 4% (48) were conformational outliers. This outcome was comparable with that of the 3CDZ structure, for which the corresponding figures were 82%, 14%, and 4% (911, 156, and 45 residues), respectively.^29^ The AlphaFold model for full-length FVIII was based on predictions using neural networks.^26^ Although the details of this modeled region are assigned as “very low confidence” in the prediction, AlphaFold showed that the B domain has a disordered outermost structure (Figure 5A-B). Its Ramachandran analysis showed that, of the 2351 residues, 75% of amino acids (1573 residues) were categorized in the “most favored” conformational regions, 15% (324) were in the “additional allowed” regions, and 9% (195) were conformational outliers.

**Figure 6.**
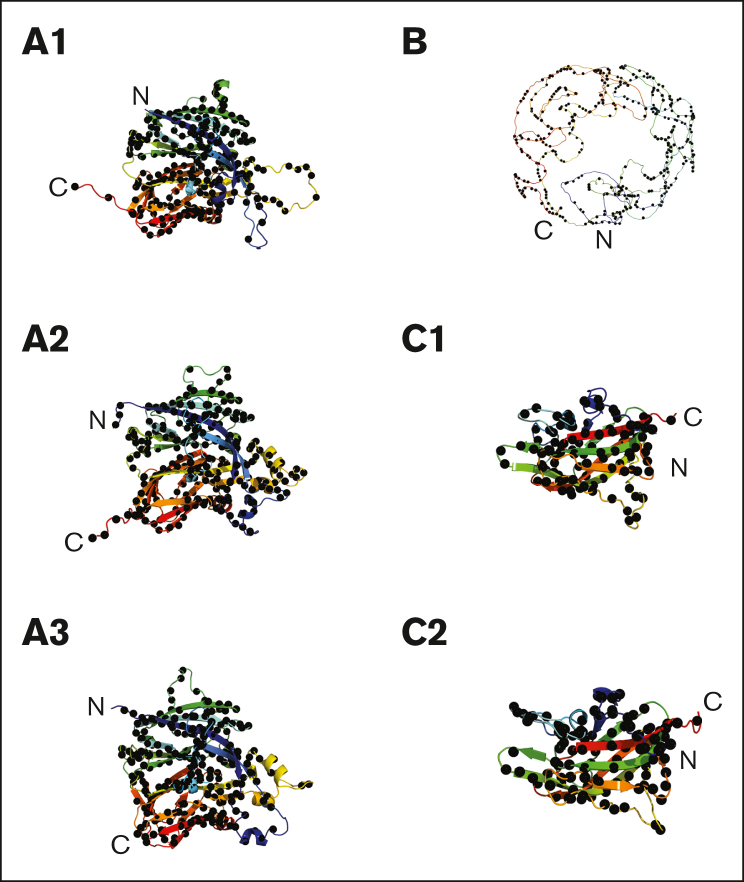
**The 6 individual FVIII domain structures and their 2863 missense variants.** The 6 structures were taken from the AlphaFold prediction. All 6 regions are shown as ribbon diagrams in rainbow colors from the N terminus (blue) to the C terminus (red). The structurally similar A1, A2, and A3 domains are shown with their secondary structure ribbons depicted in the same orientations and, similarly, the structurally similar C1 and C2 domains. The black spheres denote the missense mutations in each domain (A1, 629 variants; A2, 621 variants; A3, 537 variants; B, 483 variants; C1, 241 variants; C2, 235 variants). These variants total 2746, and the remaining 117 variants occur in the a1 linker (29 variants), the a2 linker (19 variants), the a3 linker (39 variants) and the signal peptide (29 variants). A further 1 variant was reported without information on its location. Note that the modeled structure of the B domain was predicted from AlphaFold with very low confidence and should not be overinterpreted to assume it actually surrounds the A and C domains as pictured.

The AlphaFold structure displayed the 2863 missense variants in FVIII (Figure 5C-E). Many unique variants at a given residue position showed different mild, moderate, or severe phenotypes. These multiple phenotypes are distributed in all 5 core A and C domains (Figure 5C, black spheres). The green, yellow, and red spheres showed that the 3 phenotypes were distributed throughout FVIII (Figure 5D). The 25 most common variants occurred in all 6 A, B, and C domains, with just 1 in the B domain (Figure 5E). This distribution showed that the whole FVIII structure is vulnerable to genetic variants, but there was no indication of mutational hot spots to indicate functionally important regions in FVIII. The same outcome occurred for the 2863 missense variants in the β-sheet structures of the 5 A and C domains and the disordered B domain structure (Figure 6). No variant hot spots were again seen, although the B domain showed a reduced density of variants.

**Figure 7.**
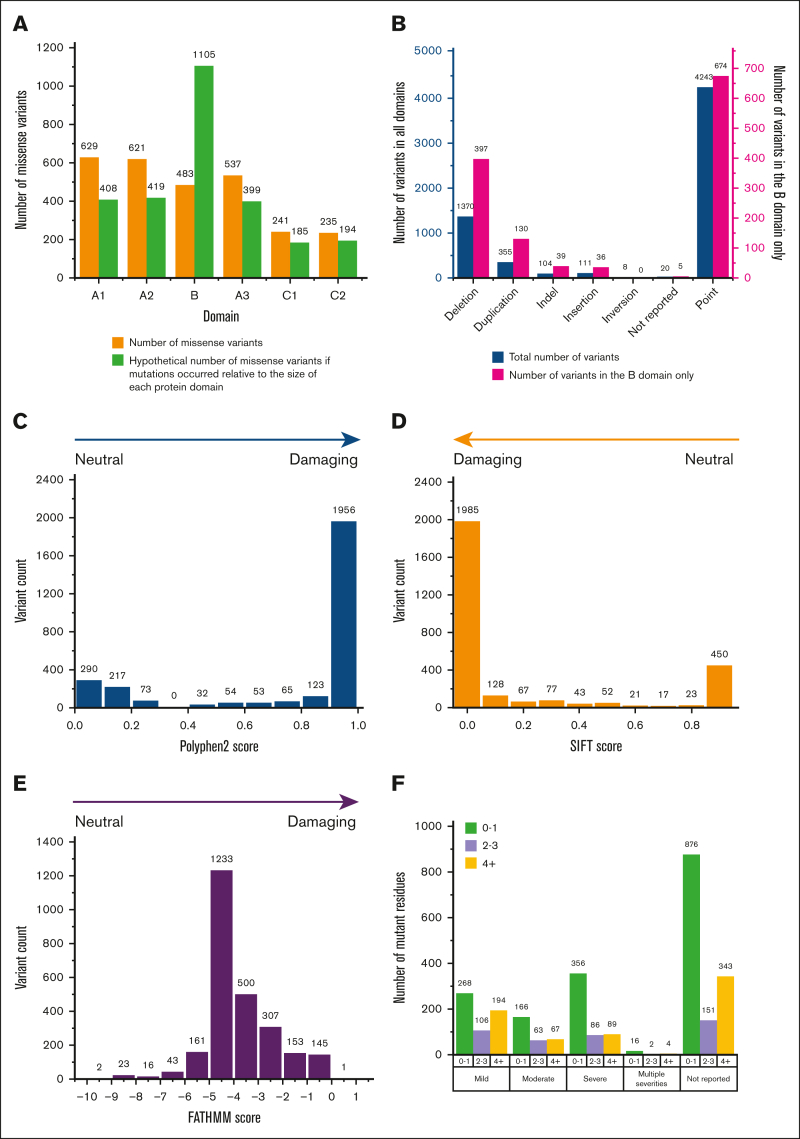
**Analyses of the variants in the 6 FVIII domains.** (A) The number of missense variants in each of the 6 FVIII domains is shown above the green bars. If the number of the 2863 missense variants is normalized in proportion to the amino acid residues present in each domain, the outcome is shown as orange bars. The missense variants in the linker regions and the signal peptide are not shown. (B) The 7 distribution types of the 1281 variants in the B domain (pink) are compared against those for all 6211 genetic variants that occur across all the protein domains in FVIII (blue). (C) The PolyPhen-2 substitution analyses predict the damaging effects of all 2863 variants from across the entire protein structure, based on the AlphaFold-predicted FVIII structure. (D) The SIFT substitution analyses predict the damaging effects of 2863 variants from across the entire protein structure based on the AlphaFold-predicted FVIII structure. (E) The FATHMM substitution analyses predict the damaging effects of 2584 missense variants from across the entire protein structure based on the AlphaFold-predicted FVIII structure. This excludes synonymous variants that are not considered by this software. (F) Accessibility analyses of 2863 missense variants in the FVIIIa crystal structure (PDB ID 6MF2). The FVIII variants were grouped by their phenotypic classification (severity) and subdivided according to the residue surface accessibility (ACC) determined using DSSP (see “Methods”). Accessibilities of 0 to 1 indicate full side chain burial; values of 2 to 3 indicate increased side-chain exposure to solvent; and values of ≥4 indicate high solvent exposure. The ACC values for the B domain (Figure 3) all indicated full surface exposures, in keeping with its predicted disordered structure.

Proportionately more variants occurred in the folded A and C domains and fewer in the B domain (Figure 7A). The increases were 54%, 48%, and 35% in the A domains and 30% and 21% in the C domains, and there was a decrease of 129% in the B domain. This difference was attributable to the greater effect of variants on the correct protein folding in the globular A and C domains, which would be less significant for the disordered B domain, which was presumed to be less susceptible to changes by variants. The B domain also showed a more varied distribution of variant types, with fewer mutational hot spots. Proportionally more deletions, duplications, indels, insertions, and point variants occurred in the B domain than other domains (Figure 7B). Fewer point variants occurred in the B domain, which can be explained by the reduced impact of the point variants on a disordered structure (Figure 3).

### Comparison of variant phenotypes with damage analyses and residue accessibilities

The observation of a missense variant in a gene is not necessarily causative of a bleeding disorder. The 2863 missense variants are too numerous to permit experimental testing of each variant through recombinant expression studies. To assist clinical interpretations, the FVIII website predicted whether a given variant would disturb the overall protein structure. The PolyPhen-2 algorithm predicted that 1956 of the 2863 missense variants (72.9%) were damaging, with scores of 0.9 to 1.0 (Figure 7C). Similarly, SIFT analysis predicted that 1985 of the missense variants (69.3%) were damaging (Figure 7D). The FATHMM analysis showed that most of the variants were damaging (Figure 7E). Such findings indicate that most of the observed FVIII variants are causative for hemophilia A. The interactive FVIII database thus provides variant-specific scores for the 2863 missense variants, providing an easy-to-use clinical support tool to clarify the significance of a variant.

To assess the existence of FVIII defects, the relationship between FVIII phenotypes and predicted solvent surface accessibilities was examined. These accessibilities of residues associated with each of the 2863 FVIII missense variants were calculated for the individually separated FVIII domains (Figure 7F). Notably, 47% of the 568 mild variants showed accessibilities of 0 or 1, highlighting their predisposition to be buried within the FVIII protein structure (Figure 7F). The same occurred for the moderate, severe, and unreported phenotypes. It can be concluded that the introduction of variants at buried locations leads to changes in the amino acid packing in the FVIII structure, which may be slight or may cause substantial regional misfolding, leading to reduced FVIII function and a bleeding phenotype.

The 6 most common variants were visually highlighted to illustrate typical effects seen with the FVIII variants (supplemental Figure 1). Synonymous variants were excluded from this diagram because they provide no structural alteration to the protein after mutation. These 6 variants have mostly mild phenotypes, but some have moderate phenotypes. Although Gly was the most commonly mutated residue in FVIII variants (Figure 4), 5 of the 6 most common nonsynonymous mutations were Arg residues (Arg612, Arg2169, Arg1985, Arg2178, and Arg2016). These occurred in 69 to 138 patients and were mutated to Cys, His, Gln, Cys, and Trp residues, respectively (supplemental Figure 1). The Arg612Cys, Arg2169His, and Arg2178Cys variants were close to the surface of FVIII, with 2 oriented into partly buried locations (Arg2169 and Arg2178) and 2 presenting an unpaired Cys residue that may interfere with the native disulfide bridge pairings within FVIII. The Arg1985Gln variant corresponded to a near-buried Arg residue located at the A2-A3 domain interface, potentially forming an ionic bridge with Glu684 to stabilize the A2-A3 contacts (supplemental Figure 1). The loss of Arg1985 would destabilize this structural interface.

## Discussion

The new genetic and structural data for FVIII within our upgraded database have the potential to transform our understanding of patients with hemophilia A, compared with our original website in 2013.^15^ It also provides further clinical insights into the occurrence of FVIII disease states and the identification of molecular targets for potential treatments. This study benefited from 3 major advances: (1) the near-tripling of the reported unique rare variants for FVIII from literature sources to a total of 6211 variants, starting from 2014 variants in 2013 (Figure 1); (2) the improved crystal structure for the A and C domains of FVIII in 2019 and the full-length FVIII protein structure available from the AlphaFold prediction^25^^,^^26^; and (3) the experience gained from our other 4 variant databases for FV, FIX, FX, and FXI.^21^, ^22^, ^23^, ^24^

Our interactive FVIII database will serve as a useful resource for clinicians and scientists to diagnose hemophilia A and predict variant effects. Database technology is able to manage the large increases in the number of known genetic variants in FVIII, when a simple spreadsheet list is no longer adequate to manage complex structural data and many variants. The website layout is designed to present both FVIII genetic and structural information, similar to that of our original FXI database and its update^18^^,^^19^^,^^22^ and our FV, FIX, and FX databases.^21^^,^^23^^,^^24^ This is illustrated using genetic and structural outputs for the most common variant Arg612Cys, for which 138 patient records exist (supplemental Figure 2). Furthermore, the amino acid Alignments tab provides further insight into the phylogenetic conservation of Arg612. It shows Arg612 aligned with 6 other mammalian species to show that this residue is fully conserved and, therefore, essential for FVIII function. On the right, the structural analysis shows that Arg612 is an exposed residue on an α-helix, and the Jmol molecular viewer shows that this is located on the A2 domain (Figure 3). Further research into FVIII using experimental studies will be key to understanding the relationship between FVIII deficiency and disease severity. The large increase in known FVIII variants, the improved 2019 crystal structure for the A and C domains in FVIII, and the 2021 AlphaFold-predicted model for full-length FVIII have greatly facilitated the analysis of the clinical effects of variants.

The mutation density within each domain is informative. The globular A1 domain has the highest mutation density in FVIII, with an average of 3.4 mutations per residue, and the other globular A2, A3, C1, and C2 domains have slightly reduced averages of 3.1, 2.8, 2.9, and 2.7 mutations per residue, respectively. In comparison, the disordered a1 linker has the lowest mutation density, with an average of just 1.2 mutations per residue, and the a2 and a3 linkers and disordered B domain also have low densities of 1.2, 1.6, and 1.4 mutations per residue, respectively. The disruption scores from the Polyphen-2, SIFT, and FATHHM predictions show that a surprisingly high proportion of the missense variants damage the FVIII protein structure (Figure 7C-E). Thus, surface accessibility changes in the packing of amino acid residues in the folded FVIII structure after residue substitution is associated with FVIII disease. If a buried variant residue is associated with FVIII disease, it is likely that the change in the residue size and/or charge will disrupt the protein structure. Most notably, we show that these 2863 missense variants are found throughout the FVIII protein structure (Figures 5 and 6), and there are no mutational hot spots that affect localized functional binding sites in FVIII. This outcome provides the clinician with guidelines on the disease significance of a new variant.

Within the family of coagulation proteins, FVIII presents unique features by virtue of its compact A and C domain structures and its large disordered B domain (Figure 5A). Coagulation FV has a similar protein structural arrangement.^44^ In contrast, FXI has a compact, well-ordered 5-domain structure (PDB ID 6I58),^45^^,^^46^ whereas FIX and FX possess extended but well-ordered 4-domain structures (PDB IDs 1PFX, 1P0S, and 1XKA).^20^^,^^47^^,^^48^ For FVIII, the AlphaFold output explicitly states that the reliability of the B domain prediction is extremely low. There is no experimentally determined B domain structure. If this did wrap itself around the A and C domains as predicted, it is hard to imagine FVIII(a) binding to VWF, its binding to membranes and other components of the tenase complex, and its accessibility for binding neutralizing anti-FVIII antibodies. Although there is currently no crystal or cryo-EM structure of full-length FVIII, the cryo-EM structures of FV and FVa showing the lack of density for almost all the B domain in the FV structure is clear evidence that the B domain is too disordered to generate interpretable density.^44^ Additionally, the extensive glycosylation of the FVIII (and FV) B domains is expected to contribute to this structural heterogeneity.^43^ Similar to FVIII, the FV, FIX, FX, and FXI missense variants are distributed throughout their protein structures, implying that these variants will perturb the correctly folded protein structure and damage their function. The interest of FVIII is that little tendencies were seen for “hot spots,” in which genetic variants accumulate in small but functionally important regions of the protein structure. Instead, many of the variants in all 5 coagulation proteins affect the surface accessibilities of amino acid residues in the overall protein structure. This observation shows that the folded structure of FVIII is susceptible to small perturbations in its entire 2351-residue protein structure, particularly where low residue surface accessibilities are involved.

Specific residue types in disease-associated variants are becoming more abundant as the number of observed genetic variants increase. The 5 charged residues Arg, Asp, Glu, His, and Lys occur 211, 179, 146, 110, and 110 times, respectively, of 2863 variants (Figure 4). This shows that changes in charged residues disproportionately feature in the variants. Charged basic residues can also be involved in FVIII interactions with negatively charged phosphatidylserine surfaces. In this study, Arg and Asp residues have been flagged as being frequent origin-of-disease–associated variants in FVIII (Figure 4). Furthermore, Arg comprised 5 of the 6 most common variants seen in FVIII (supplemental Figure 1). Being the most basic of the 20 common amino acids, Arg residues are important for the stability of ionic interactions at protein surfaces, whether this be with the binding of substrates or forming interdomain interactions.

As a postscript to this study, we investigate whether the occurrence of variants is similar in FVIII with those in the structurally similar FV protein.^44^ The percentage distribution of the variants between the 6 domains A1, A2, B, A3, C1, and C2 show that the variants are distributed among all 6 domains in both proteins. However, there are proportionately more variants in the A1 and B domains of FVIII than FV (supplemental Figure 3). Conversely, there are proportionately more variants in the A3 domain of FV than FVIII. The predominance of missense variants at positively charged Arg and negatively charged Asp seen above for FVIII was also found in FV. The spatial distribution of the variants in FVIII and FV also appear similar, further supporting the conclusion that there are no hot spots or clusters of variants (supplemental Figure 4). Thus, the earlier FV study acts as an independent verification of our FVIII study.

Conflict-of-interest disclosure: The authors declare no competing financial interests.
