# Supplementary material for: Analyzing 6211 unique variants in the upgraded interactive FVIII web database reveals novel insights into hemophilia A
Source: Blood Vessel Thromb Hemost. 2025 Jan 21;2(3):100053. doi: 10.1016/j.bvth.2025.100053 (PMC12320434; doi:10.1016/j.bvth.2025.100053)
Supplement: Supplemental Figures [file BVTH_VTH-2024-000215-mmc1.pdf]

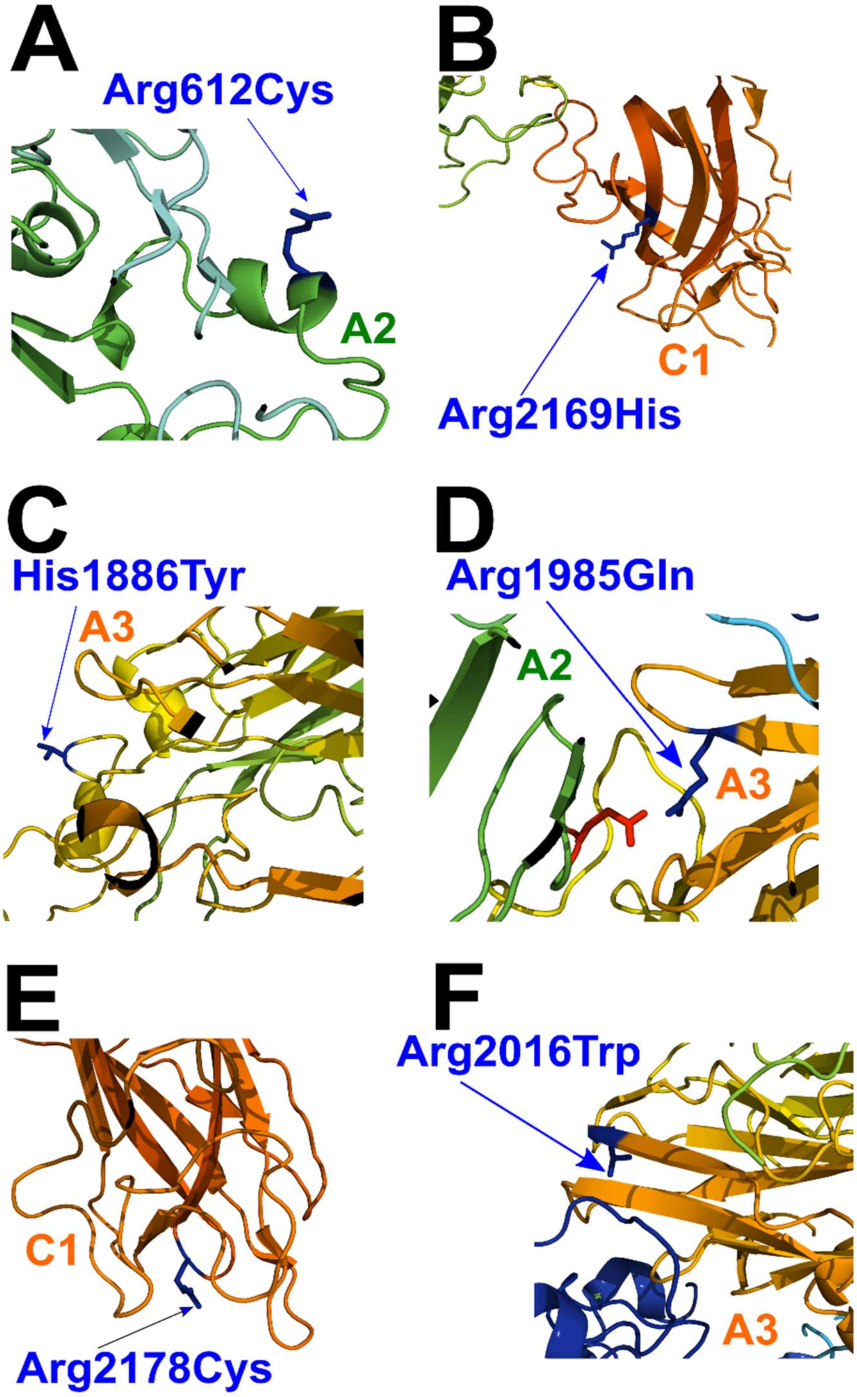

Supplementary Figure S1: Molecular graphic representation of six residues within the FVIIIa protein structure.

The six panels highlight the six most-commonly occurring synonymous variants in the FVIIIa crystal structure (PDB ID: 3CDZ). The 3CDZ structure was selected for modelling in this study because the 3CDZ structure is an unbound form of FVIII, which better represents the native conformation of protein in solution. The other structures with PDB IDs of 6MF2 and 7KWO show similar structures. For each, the residue of interest in the native FVIIIa protein are shown. Panels A, B, C, D, E and F highlight the wildtype residues Arg612 (A2 domain; 138 patients), Arg2169 (C1 domain; 103 patients), Gly1238 (B domain; 91 patients), His1886 (A3 domain; 83 patients), Arg1985 (A3 domain; 69 patients), and Arg2178 (C1 domain; 69 patients). All residue numbering is given in HGVS format.

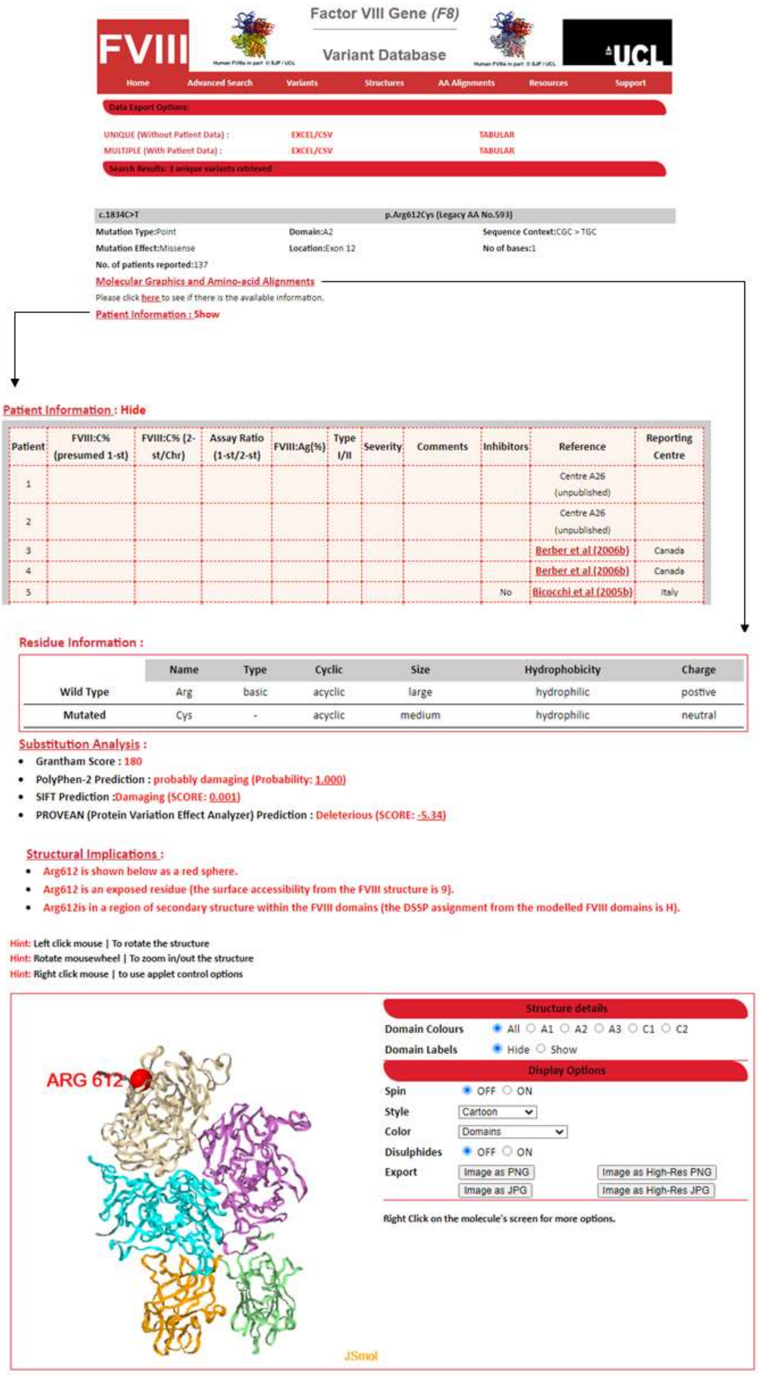

Supplementary Figure S2: Screenshots of the upgraded FVIII web site to illustrate the analysis made for the common Arg612Cys variant. The upper panel displays the output when the Arg612 residue is inputted on the home page of the interactive web site. By clicking "Show" on the patient information, the lower left panel lists genetic information for patients reported with the Arg612Cys variant, of which five records are visible, together with the source of the patient record. Clicking "HERE" on the structural interpretation gives the image shown on the bottom right panel. This assesses the buried or exposed accessibility of the variant and its location in the FXI protein structure. A JMol view of the FVIII structure is displayed that can be rotated and zoomed into as desired. Four substitution analyses to predict the damaging effects of each missense variant is provided to facilitate clinical diagnosis.

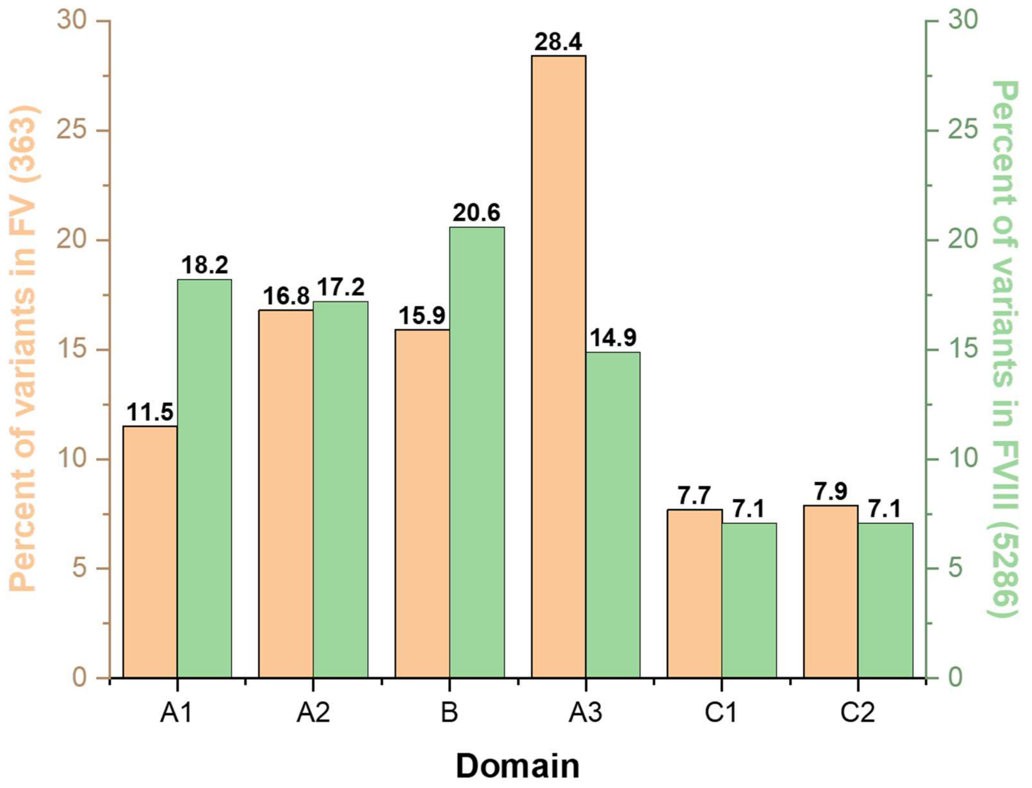

Supplementary Figure S3: Percentage distribution of variants in the six FV and FVIII domains. The percentage of the 363 and 5286 total variants found in each of the six core domains of FV (orange) and FVIII (green) is shown above the bars. The variants that occur in the UTRs, linker regions, and the signal peptides are not shown.

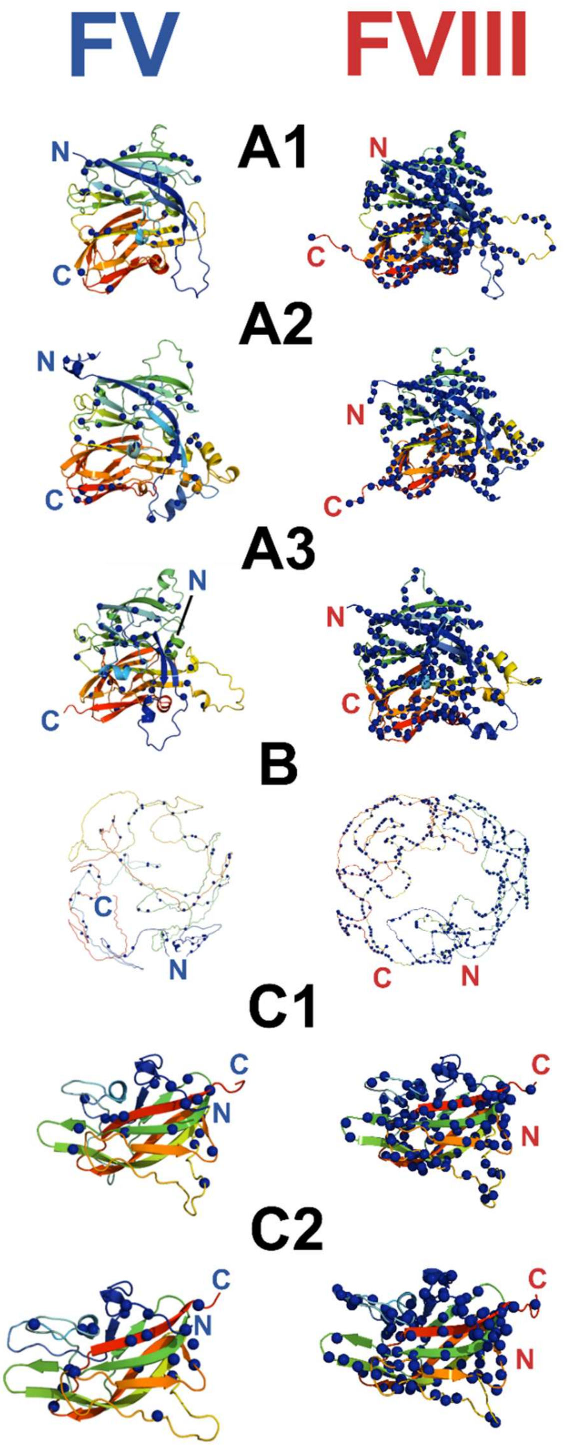

Supplementary Figure S4: The missense variants are shown for the six FV (left) and FVIII (right) domain structures. All six domains are shown as ribbon diagrams that are colour-coded in rainbow colours from the N-terminus (blue) to the C-terminus (red) for clarity. The structurally-similar A1, A2 and A3 domains are shown with their secondary structure ribbons depicted in the same orientations, and likewise the structurally-similar C1 and C2 domains. The blue spheres denote the missense mutations in each domain.
